# Cardiac Signals Facilitate the Breakthrough to Awareness of Emotional Stimuli

**DOI:** 10.1111/psyp.70168

**Published:** 2025-10-22

**Authors:** Ozan Cem Ozturk, Jessica McFadyen, Ruben T. Azevedo

**Affiliations:** ^1^ School of Psychology University of Kent Canterbury UK; ^2^ Limbic Ltd London UK

**Keywords:** cardiac cycle, continuous flash suppression, emotion, interoception, unconscious

## Abstract

The brain continuously integrates interoceptive signals—such as those arising from cardiac afferents—with sensory input to guide perception, emotion and awareness. Previous research has demonstrated that the timing of external stimuli relative to the cardiac cycle influences perceptual and cognitive processes. However, it remains unclear whether cardiac signals facilitate the access of emotional visual stimuli to conscious awareness. Here, we used a continuous flash suppression (CFS) paradigm to investigate whether the breakthrough of fearful and neutral faces to awareness is modulated by cardiac cycle phase. Fearful and neutral faces were presented to the non‐dominant eye in synchrony with participants' heartbeats—either during estimated‐cortical systole (ec‐systole) or diastole (ec‐diastole)—while dynamic Mondrian patterns suppressed visibility in the dominant eye. Results showed that fearful faces presented during estimated‐cortical systole (ec‐systole) broke through suppression faster and after fewer heartbeat‐synchronized presentations than those presented during ec‐diastole, suggesting facilitated processing. No significant cardiac modulation was found for neutral faces or in emotion discrimination accuracy, confidence, or response bias. These findings demonstrate that cardiac afferent signals selectively enhance the perceptual salience of motivationally salient (e.g., threat signaling) stimuli, promoting earlier access to consciousness. This study extends prior work by showing that cardiac influences on emotion processing operate even at early, preconscious stages of visual perception.

## Impact Statement

This study shows that the timing of visual stimuli relative to the cardiac cycle modulates conscious perception of emotional faces. Fearful, but not neutral, faces presented during systole broke through visual suppression more rapidly, suggesting that interoceptive signals enhance the salience of emotionally relevant stimuli. These findings show how body‐to‐brain signals can shape conscious visual awareness at early processing stages and contribute to a growing understanding of how physiological arousal modulates sensory and affective perception.

## 1 Introduction

The visual system is overloaded with more information than it can fully process at an optimal time, requiring a selection mechanism that determines which stimuli receive deeper processing and reach conscious awareness (Pourtois et al. 2013). This selection mechanism goes beyond the visual system and is shaped by an interplay between bottom‐up factors, such as stimulus properties and top‐down influences, including context and motivation. Research has consistently shown that emotionally salient stimuli, especially if they are threat‐related, have privileged access to processing resources, even prior to any conscious awareness. Subcortical structures like the amygdala, pulvinar and superior colliculus play a crucial role in this process by filtering sensory input, directing attention and even contributing to early visual processing, which may be particularly important for salient or threatening stimuli (Tamietto and de Gelder 2010; McFadyen et al. 2020; Diano et al. 2017).

It has been proposed that physiological arousal may be a key modulator of this subcortical network enhancing the processing of salient or emotionally significant stimuli to prepare the organism for adaptive responses (Garfinkel and Critchley 2016; McFadyen et al. 2020; Diano et al. 2017). While the precise mechanisms through which arousal shapes visual perception remain poorly understood, there is growing consensus that the brain's continuous and dynamic representation of internal bodily states—such as those conveyed by cardiac afferent signals—plays a pivotal role in shaping emotions and forming the foundation of the self, underpinning subjective experience (Ainley et al. 2016; Garfinkel and Critchley 2016; Nord and Garfinkel 2022; Engelen et al. 2023; Skora et al. 2022). This perspective emphasizes the importance of interoception—the processing and awareness of internal bodily signals—and more broadly of brain–body communication, whereby bodily states continuously interact with neural processes. Within this framework, the human brain continuously integrates two distinct information streams to create ‘a unified conscious experience’, both of which influence processes such as memory, mood, expectations, perception and attention (Adolfi et al. 2017; Berntson and Khalsa 2021). Top‐down processing involves cognitive modulation where prior knowledge, experiences and expectations shape the interpretation of incoming signals. In parallel, bottom‐up processing that is driven by sensory inputs (e.g., cardiac, gastric or respiratory signals) is transmitted hierarchically to the brain, contributing to shape perception and influence cognitive processes (Adamic et al. 2024). Together, these streams ensure that the brain dynamically adjusts perception and awareness (Nord et al. 2021). However, due to the continuous interplay between these two streams of processing, it remains relatively challenging to experimentally manipulate and test the influence of bottom‐up interoceptive sensory information on cognition.

To overcome this challenge, researchers have recently developed experimental paradigms that align stimulus presentation with different phases of physiological cycles (e.g., cardiac, respiratory) to investigate how interoceptive signals influence psychological processes (Aspell et al. 2013; Garfinkel and Critchley 2016; Engelen et al. 2023; Monti et al. 2020). In the cardiac domain, research has shown cardiac cycle influences the processing of, for example, somatosensory (Edwards et al. 2009; Galvez‐Pol et al. 2022; Motyka et al. 2019), painful (Edwards et al. 2001; McIntyre et al. 2006) and visual stimuli (Garfinkel et al. 2014; Pramme et al. 2016; Azevedo et al. 2023; Ambrosini et al. 2019). While the mechanisms underlying the observed effects in these paradigms are still a matter of debate (Garfinkel and Critchley 2016; Skora et al. 2022; Engelen et al. 2023), a prominent proposal is that these are mediated by the phasic firing of arterial baroreceptors—pressure and stretch sensors that detect fluctuations in blood pressure and signal them to the brain during the systolic phase of each heartbeat (for a discussion see Garfinkel and Critchley 2016). The basic premise is that the processing of exteroceptive stimuli is modulated while the brain is processing these cardiovascular afferent signals (we will call this period estimated‐cortical systole (ec‐Systole; see Caparco et al. 2024)) compared to the period in which their representation in the brain is minimal (estimated‐cortical diastole; ec‐Diastole). The proposal is that these short‐term and preconscious fluctuations in baroreceptor activity may create a physiological context that modulates body–brain interactions, influencing the processing and neurophysiological responses to sensory stimuli (Garfinkel and Critchley 2016).

Importantly, however, such modulations depend on the overall context and stimuli type. Most notably, while there is an inhibition in the processing of weak sensory stimuli, for example, touch (Galvez‐Pol et al. 2022; Motyka et al. 2019) and painful stimulation (Edwards et al. 2001; McIntyre et al. 2006) during ec‐Systole, the processing of attention‐orienting or motivationally relevant visual stimuli seems to be enhanced during this same period (Garfinkel et al. 2014; Azevedo et al. 2017, 2018; Mizuhara and Nittono 2023; Ambrosini et al. 2019). In the context of threat processing, fearful faces presented at ec‐systole (vs. ec‐diastole) are perceived as more intense, capture greater attentional resources in a dot‐probe task and are more easily detected among distractors in an attentional blink paradigm (Garfinkel et al. 2014; Azevedo et al. 2018; Mizuhara and Nittono 2023). It has been argued that in contexts requiring heightened alertness to threat‐related stimuli, an increased cortical representation of cardiac signals may amplify the salience of these cues (Garfinkel and Critchley 2016), an effect likely mediated by subcortical regions involved in arousal and salience processing, such as the amygdala, periaqueductal gray and insular cortex (Garfinkel et al. 2014; Gray et al. 2009).

While evidence continues to accumulate on the enhancement effects of cardiac signals on salience and attentional networks, there are still gaps in knowledge in terms of how these neurocognitive processes impact the sensory processing of visual stimuli. One of these questions relates to whether interoceptive signals contribute to the prioritized or facilitated access to consciousness of emotional visual stimuli. Previous research (Salomon et al. 2016, 2018) investigated this by entraining simple visual stimuli (e.g., a dot) to participants' heartbeats in a continuous flash suppression (CFS) paradigm, where dynamic, high‐contrast patterns (such as flashing colored shapes or noise) are presented to one eye—usually the dominant eye—while a static image (e.g., a face or object) is presented to the other eye (e.g., Tsuchiya and Koch 2005; Costello et al. 2009; Gayet et al. 2014). The flashing patterns dominate visual perception, effectively suppressing the static image from reaching conscious awareness for several seconds. Using this approach, Salomon et al. (2016, 2018) demonstrated that simple visual stimuli synchronized with the participants' heartbeats took longer to break through to conscious awareness, a suppression mediated by activity in the insular cortex. These findings suggest cardiac‐mediated inhibition in the processing of simple stimuli at early visual processing stages. However, they do not inform on how cardiac afferent signals modulate the breakthrough to awareness of motivationally salient stimuli. A wealth of research has shown facilitated processing of emotional stimuli, such as those signaling threat, presented under CFS (Oliver et al. 2015; Vieira et al. 2017; McFadyen et al. 2022; Pelliet et al. 2025), demonstrating the usefulness of this paradigm to study the privileged access of affective information to conscious awareness.

Whereas previous studies suggest cardiac phase‐related suppression for low‐salience stimuli, it remains unclear whether emotionally salient stimuli—such as fearful faces—might instead be facilitated under similar conditions. To study this, we used a CFS paradigm where we presented rapidly changing Mondrian patterns to the dominant eye and flashing images—either fearful or neutral faces—to the non‐dominant eye. Face presentations flashed in synchrony with the participants' heartbeats, either at ec‐systole or ec‐diastole. In this context of detection of motivationally relevant (i.e., social) stimuli, we predicted an earlier breakthrough to awareness of stimuli synchronized with participants' cardiac ec‐systole (vs ec‐diastole). In line with previous findings of cardiac cycle enhancement in the processing of threat‐signaling stimuli, we expected this effect to be particularly strong for fearful faces. As explorative analyses, we also tested if the cardiac cycle modulates the ability and confidence to discriminate the expressions of the perceived faces or response biases, that is, the tendency to answer ‘fear’ over ‘neutral’.

## 2 Methods

### 2.1 Participants

Our sample comprises a total of 60 participants (49 females; 11 males; mean age = 20.27; sd = 3.6) with no history of cardiovascular or neurological disorders. Data from three additional participants was also collected but not analyzed due to difficulties in following task instructions. A power analysis based on Salomon et al. (2016)'s effect size (Cohen's *d* = 0.38) revealed a minimal sample size of 57 to detect a cardiac cycle effect with a power of 0.80, which is in line with other similar studies (Veillette et al. 2024). The study received ethical approval from the University of Kent School of Psychology's ethics committee, and written informed consent was obtained from all participants.

### 2.2 Stimuli

**FIGURE 1:**
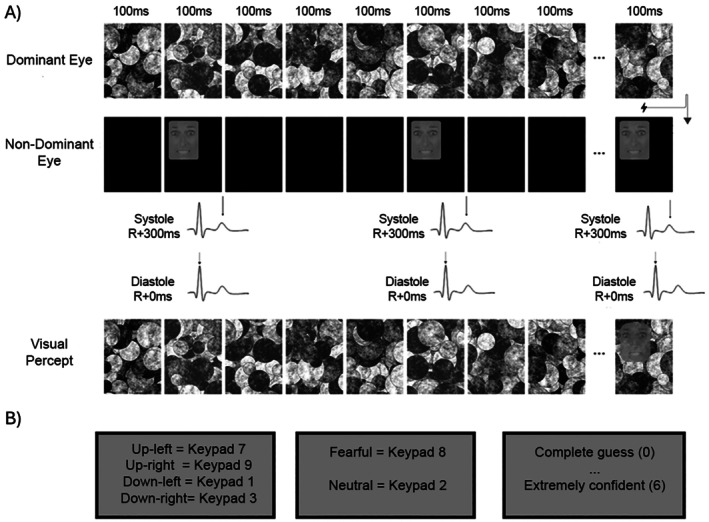
Illustration of a single trial in this experiment. (A) Different Mondrian images are presented to the dominant eye each 100 ms without break. A face, either neutral or fearful, is shown to the non‐dominant eye in synchrony with the participant's cardiac cycle, either during ec‐systole or ec‐diastole, in one of four possible quadrants of the designated screen area, for 100 ms. Stimuli presentation ends when the participant presses the “spacebar” to indicate they have perceived a face or after 10s. (B) Then, participants answer with key presses: (i) in which quadrant they think the face was presented, (ii) whether it had neutral or fearful expression, (iii) and how confident are they in their facial discrimination decision (on a scale 0–6).

Stimuli consisted of black and white faces of male and female actors, from different ethnicities, posing either with a neutral or fearful facial expression. These photos (170 × 220 pixels) were taken and adapted from the Chicago Face Database stimuli set (Ma et al. 2021) and comprised 26 different individuals. Twenty different stimuli were used for neutral expressions and 26 for fearful expressions. Oval‐shaped gray contours were superimposed on the photos to remove features such as hair, ears and neck (Figure 1). Stimuli were matched for mean luminance and contrast using the SHINE toolbox for Matlab (Willenbockel et al. 2010). We used Mondrian patterns as mask stimuli. There were 20 different black and white images (520 × 720 pixels) created using the software CFS_Crafter (Wang et al. 2023). The Mondrians were larger than the face stimuli to allow presenting faces at the four different quadrants within the mask (see below).

### 2.3 Procedure

After determining the participant's dominant eye using the Miles test and fitting the ECG electrodes, participants were seated comfortably ~60 cm from a computer screen (1920 × 1080 pixels at 60 Hz) with their chins resting on a chinrest with prism glasses attached to it. While the glasses ensured participants' left and right eyes only received information presented on the left and right sides of the screen, respectively, individual calibration was required to ensure stimuli presented to both eyes were aligned, that is, they were perceived in the same location. To do that, participants adjusted, using the left and right cursor keys of the keyboard, the position of a small rectangle, the size of the face stimuli, until it aligned with one of the borders of a larger rectangle, the size of the Mondrians. They repeated this for the four quadrants (up‐left; up‐right; down‐left; down‐right). In the main task, the borders of the face stimuli were presented 60 pixels inside the Mondrian image to prevent any eventual small misalignments from leading to immediate face visibility.

Next, because there are known individual differences in how easily/quickly stimuli presented to the non‐dominant eye breakthrough awareness in CFS, we calibrated the level of contrast of the target images to be presented to the non‐dominant eye. We wanted to make sure that each participant, on average, would need a few presentations (i.e., flashes) of the stimuli for it to be consciously perceived. The calibration followed a similar format to the experimental task, and the same stimuli were used to ensure the validity of the calibration and serve as practice. Specifically, participants were presented with Mondrian images changing every 100 ms to their dominant eye and faces flashing for 100 ms in synchrony with their own heartbeats (either at ec‐Systole or ec‐Diastole) to their non‐dominant eye. The participants' task was to press the spacebar as soon as they perceived the face breaking through the CFS. Stimuli presentation terminated with the key press or 10s after trial onset. Then, participants were asked to indicate with key presses: (i) in which of the 4 quadrants of the Mondrian image the face was presented (this was to promote spatial vigilance and orienting responses); (ii) whether it had a neutral or fearful facial expression; (iii) and how confident they were on the emotion judgment on a 6‐point Likert scale where 0 meant “complete guess” and 6 “extremely confident” (see Figure 1). The calibration started with a contrast value of 0.2, where 0 is fully transparent, that is, no stimuli, and 1 is full contrast, that is, original image. This starting value was determined based on pilot data as likely to lead to average breakthrough times between 3 s and 7 s. The calibration then followed a staircase procedure according to the following conditions: if the participant did not report breakthrough to awareness, contrast was increased by 0.03; conversely, if the reaction time in the preceding trials was lower than 3.5 s, contrast was decreased by 0.015. Moreover, we estimated the moving average of reaction times over the 4 preceding trials: if it was lower than 5 s, contrast was decreased by 0.03; if it was higher than 8.5 s, contrast was increased by 0.015; if it was between 5 s and 8.5 s (or a max of 30 trials was reached), the calibration procedure ended and the last contrast value was adopted. Of note, because the main focus of the study was on breakthrough times, the accuracy in face identification was not considered for calibration purposes.

The main experimental task was identical to the calibration except for the contrast of the target image. In the first presentation (i.e., first flash) the image had the contrast value determined during the calibration but this increased after each presentation by 3.5% of the original value. The task comprised total of 160 trials (i.e., 40 per each Cardiac cycle × Emotion condition) separated by a random inter‐trial interval between 2000 and 4250 ms. Participants were given three breaks to rest, and the task had an approximate duration of 40–45 min.

### 2.4 Stimuli Synchronization

ECG electrodes were attached to the participants' upper chest and lower back in a modified Lead‐II position. The ECG signal was recorded with a BIOPAC MP150 hardware and Acknowledge 3.5 software (https://www.biopac.com/) with a sampling rate of 1000 Hz. Real‐time heartbeat detection was achieved with the DTU hardware module (https://www.biopac.com/product/digital‐trigger‐unit‐for‐mri‐gating/), which uses an individually tailored threshold method to detect the ECG's R‐peak (i.e., when the ECG trace exceeds the threshold value) and deliver TTL pulses with negligible delay. Custom‐built code in Matlab was used to receive these TTL pulses and present face stimuli either 300 ms after the ECG's R‐peak (ec‐systole) or when the heartbeats were detected (i.e., *R* + 0 ms; ec‐diastole; cf. Garfinkel et al. 2014; Li et al. 2020).

### 2.5 Data Analyses

The study followed a fully within‐subjects design with two independent variables: Cardiac Cycle (ec‐Systole; ec‐Diastole) and Facial Expression (Neutral; Emotion). To test if the cardiac cycle influences the breakthrough to awareness in CFS, we had two DVs: Reaction Times (RTs; i.e., how long it took for participants to report awareness of the stimulus) and the number of heartbeats (nHBs; i.e., corresponding to face presentations) necessary for the stimulus to become visible. We also analyzed accuracy and confidence in the identification of the facial expression and carried out signal detection theory analyses (Macmillan and Creelman 1991) to explore differences in sensitivity (d′) or response bias (*c*) as a function of the cardiac cycle. These measures explore differences in the ability to discriminate the facial expressions and tendency to provide one particular type of answer, respectively, at different phases of the cardiac cycle. Larger *d’* shows improved ability to discriminate between the two facial expressions, and positive *c* values reflect the tendency to answer ‘neutral’ over ‘fear’ whereas negative *c* values indicate the opposite tendency.

In average, participants correctly identified the correct location of faces in 83.8% (sd = 16.0%) of trials. There was no difference between cardiac cycle conditions (*p* = 0.32), but participants made more errors for fearful faces (*p* = 0.029). Trials with incorrect or missed identification of heartbeats, leading to additional or fewer presentations of face stimuli, respectively, were identified as follows (cf Kemper et al. 2007): (i) any interbeat interval (i.e., time between consecutive face presentations) longer than 1300 ms or shorter than 400 ms, corresponding to ~46 and 150 bpm, respectively; any interbeat interval greater or smaller than 5 deviations from the mean of all interbeat intervals (across trials) for that participant. These trials (*n* = 207, mean = 3.45, sd = 5.32) were excluded from analyses. For the analyses of RTs and nHBs, we also excluded trials with no key press (mean percentage = 16%, sd = 17.6%). There were more neutral (mean = 4.48, sd = 4.69) than fearful (mean = 3.5, sd = 4.26) face trials with no key press (*t* = 4.36, *p* < 0.001), but no difference as a function of cardiac cycle (*p* > 0.05).

The remaining trial‐by‐trial data was entered on generalized linear mixed models, using the {*lme4}* package (Bates et al. 2015) in R (https://posit.co/). Cardiac Cycle, Facial Expressions and their interaction were entered as fixed effects, and trial number and the Location where faces were presented were used as covariates. Models with nHBs or Confidence as DV were fitted using a Poisson probability distribution, the preferred method to analyze count data (Yadav et al. 2021). RTs data were fitted with a Gamma log link distribution (Lo and Andrews 2015), and emotion accuracy with a binomial distribution. Participant code and picture code were added as random intercepts. Predictors were also modeled as random slopes, first tried over participants and then pictures when it did not lead to singularity or convergence issues. The final models can be found in Table 1. Statistical significance was determined using the *Anova()* function of the *{car}* package (Fox and Weisberg 2019). Planned post hoc comparisons were carried out using the emmeans() function of the {emmeans} package (Lenth 2025) to test differences between ec‐Systole and ec‐Diastole. Given the planned and small number of post hoc tests, no correction for multiple comparisons was applied. Pairwise *t*‐tests were performed to analyze changes in d' and c as a function of the cardiac cycle. Data and scripts used for the analyses can be found here: https://osf.io/f6btp/.

**TABLE 1:** Final models for the generalized linear mixed model analyses for each DV.

DV	Model
RT	glmer(RT ~ FacialExpression * CardiacCycle + Location + TrialNumber + (1 + FacialExpression + Location | Participant) + (1 + TrialNumber | PictureCode), family = Gamma(link = “log”), data = CFSrt)
nHB	glmer(nHB ~ FacialExpression * CardiacCycle + Location + TrialNumber + (1 + FacialExpression + Location + TrialNumber | Participant) + (1 + TrialNumber | PictureCode), family = poisson(link = log), data = CFSrt)
Accuracy	glmer(Accuracy ~ FacialExpression * CardiacCycle + Location + TrialNumber + (1 + FacialExpression + TrialNumber | Participant) + (1 + Location | PictureCode), family = ‘binomial’, data = CFS)
Confidence	glmer(Confidence ~ FacialExpression * CardiacCycle + Location + TrialNumber + (1 + FacialExpression + TrialNumber | Participant) + (1 | PictureCode), family = poisson(link = log), data = CFS)

## 3 Results

**FIGURE 2:**
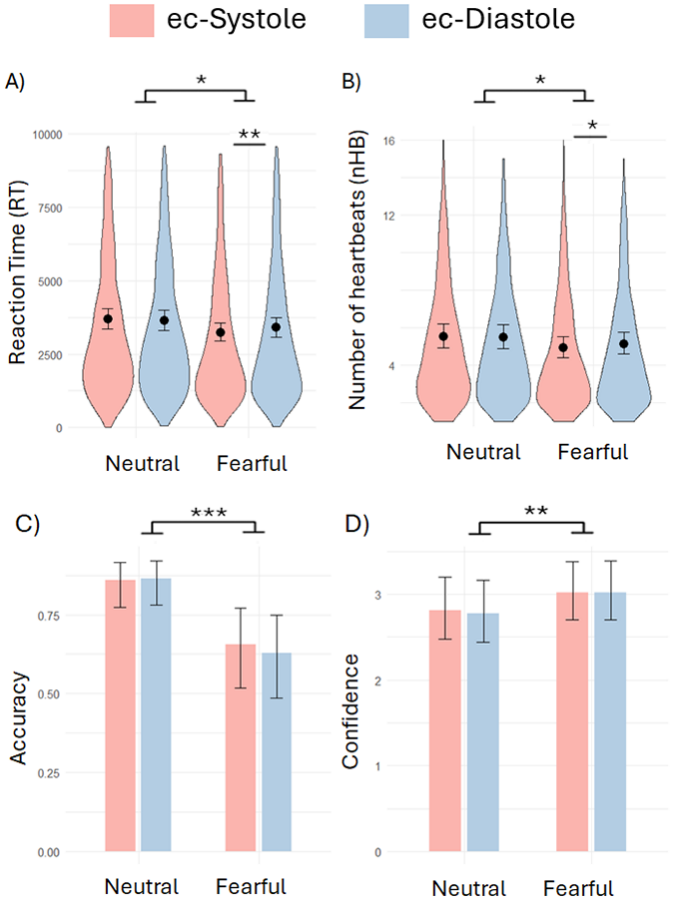
Behavioral results. Violin plots depicting the distribution of responses in all trials and model adjusted means of (A) Reaction times (RT) in ms and (B) the number of heartbeats (nHB) necessary for the stimuli to breakthrough to awareness in each condition. In brief, the presentation of fearful faces, especially those presented at ec‐systole (vs. ec‐diastole), was associated with shorter RTs and required less presentations (i.e., nHB) to breakthrough to awareness. Model adjusted means of (C) Accuracy and (D) Confidence in the discrimination of facial expressions, showing lower accuracy but greater confidence in the identification of fearful (vs. neutral) faces. *p < 0.05, **p < 0.01, ***p < 0.001.

In the regression model on RTs, we found a significant main effect of Facial Expression (*χ* ^2^ = 4.08, *p* = 0.043; Figure 2A), confirming the known facilitated processing of fearful faces in CFS. Interestingly, the interaction between Cardiac Cycle and Facial Expression was also significant (*χ* ^2^ = 7.02, *p* = 0.008). Post hoc analyses revealed that fearful (z ratio = −2.76, *p* = 0.006) but not neutral (z ratio = 0.96, *p* = 0.34) expressions presented at ec‐Systole (vs. Diastole) required less time to breakthrough to awareness (Figure 2A). The main effect of Cardiac Cycle (*χ* ^2^ = 1.53, *p* = 0.21) was not significant. We also observed a main effect of Location (*χ* ^2^ = 18.997, *p* < 0.001), which could be explained by quicker RTs when faces were presented in the upper‐right quadrant compared to all the other locations (z ratios > 3.20 ps < 0.015; all other ps > 0.21). Finally, a significant main effect of trial number was observed (*χ* ^2^ = 98.63, *p* < 0.001), reflecting the known progressive weakening of binocular suppression power over time.

In line with the results observed for RTs, in the model predicting the number of heartbeats (nHB) necessary for the stimuli to breakthrough awareness we found a significant main effect of Facial Expression (*χ* ^2^ = 6.38, *p* = 0.011; Figure 2B) and a significant interaction between Cardiac Cycle and Facial Expression (*χ* ^2^ = 5.82, *p* = 0.016). Post hoc analyses confirmed that fearful (z ratio = −2.93, *p* = 0.003) but not neutral (z ratio = 0.39, *p* = 0.70) expressions presented at ec‐Systole (vs Diastole) required fewer presentations to breakthrough to awareness (Figure 2B). The main effect of Cardiac Cycle (*χ* ^2^ = 2.94, *p* = 0.086) was not significant. Finally, the main effects of trial number (*χ* ^2^ = 41.11, *p* < 0.001) and Location (*χ* ^2^ = 24.90, *p* < 0.001) were also confirmed.

The regression on Accuracy revealed poorer accuracy (*χ* ^2^ = 17.11, *p* < 0.001) in the identification of fearful expressions. Neither the main effect of Cardiac Cycle (*χ* ^2^ = 0.62, *p* = 0.43) nor its interaction with Facial Expression (χ2 = 1.92, *p* = 0.16) was found to be significant (Figure 2C). Results also showed a significant main effect of trial number (*χ* ^2^ = 12.62, *p* < 0.001), with accuracy increasing throughout the task, but no effect of Location (*χ* ^2^ = 3.45, *p* = 0.33). The analyses on the signal detection theory indices revealed neither a cardiac cycle effect on *d’* (mean systole = 2.46, sd = 1.5; mean diastole = 2.44, sd = 1.50; *t*(1, 59) = 0.26, *p* = 0.80) nor on *c* (mean systole = 0.30, sd = 0.35; mean diastole = 0.30, sd = 0.34; *t*(1, 59) = −0.059, *p* = 0.95).

In what regards Confidence, participants showed greater confidence (*χ* ^2^ = 6.75, *p* = 0.009) in the identification of fearful expressions, but neither the main effect of Cardiac Cycle (*χ* ^2^ = 0.34, *p* = 0.56) nor its interaction with Facial Expression (*χ* ^2^ = 0.37, *p* = 0.4) were found to be significant (Figure 2D). Both the main effects of trial number (*χ* ^2^ = 15.47, *p* < 0.001) and Location were significant (*χ* ^2^ = 16.93, *p* < 0.001). Post hoc analysis revealed higher confidence in the upper‐right quadrant versus upper‐left (*χ* ^2^ = 4.09, *p* < 0.001) and down‐left (*χ* ^2^ = 2.26, *p* = 0.023) quadrants, and lower confidence in the upper‐left versus lower‐right (*χ* ^2^ = −2.16, *p* = 0.031) quadrant.

## 4 Discussion

In this study, we sought to investigate if cardiac afferent signals contribute to the facilitated access to consciousness of emotional visual stimuli. For that, we presented faces with neutral or fearful expressions in synchrony with the participants' heartbeats in a CFS paradigm. Our results confirmed our prediction of facilitated breakthrough to awareness of salient stimuli presented in synchrony with participants' heartbeats. Specifically, fearful faces presented during ec‐systole (vs ec‐diastole) required less time and fewer presentations to be consciously perceived. No effects were found on the tendency (i.e., response bias) or ability (i.e., accuracy or confidence) to identify the facial expressions. This study shows how spontaneous fluctuations in (the processing of) afferent physiological signals interact with early visual perception to facilitate access to emotional stimuli.

Previous research using CFS found longer reaction times for stimuli presented in synchrony with the participants' heartbeats (vs faster or slower) independently of cardiac phase (Salomon et al. 2016). The authors interpreted these results as an inhibition in the processing of exteroceptive stimuli concurrent with ongoing heartbeats. They proposed that this is a by‐product of the suppression of the sensory consequences of cardiac signals. That is, as the blood travels through veins and arteries, it induces an array of sensory events that need to be canceled out by the brain, similarly to the suppression of sensations associated with self‐generated actions (Shergill et al. 2013). The result is a general suppression in cognitive and sensory processing (Lacey and Lacey 1978; Salomon et al. 2016). This idea is in line with substantive research showing cardiac cycle inhibition in several different processes, such as memory (Garfinkel et al. 2013), pain (Edwards et al. 2001; McIntyre et al. 2006) and weak tactile stimulation (Galvez‐Pol et al. 2022; Motyka et al. 2019).

However, it does not explain the observed enhanced performance in other tasks (e.g., Pramme et al. 2016; Marshall et al. 2024) or the up‐regulation in the processing of salient social stimuli, most notably, threat signaling stimuli at ec‐systole (e.g., Garfinkel et al. 2014; Azevedo et al. 2017; Mizuhara and Nittono 2023). It has been proposed that in moments requiring heightened alertness, an increased cortical representation of cardiac signals may amplify the salience of motivationally relevant cues and inhibit that of weak or less relevant stimuli (Garfinkel and Critchley 2016). So far, research has shown that the cardiac cycle affects mostly the attention grabbing and orienting power of fearful faces, as evidenced in attentional blink (Garfinkel et al. 2014), dot‐probe (Azevedo et al. 2018), Go/No‐Go (Yang et al. 2023) and implicit racial bias (Azevedo et al. 2017) tasks. Our study expands these observations by showing cardiac cycle enhancement at early stages of visual processing in conditions of sensory ‘unawareness’ (Tamietto and de Gelder 2010).

Binocular rivalry paradigms, such as the CFS, interfere directly with activity in the ventral occipitotemporal cortex, known to be crucial for visual awareness. This contrasts with paradigms engaging attentional processes, such as the attentional blink, which may suppress activity in visual cortices through top‐down attentional influences, leading to ‘attentional unawareness’ (Tamietto and de Gelder 2010; McFadyen et al. 2020). However, in both approaches, emotional stimuli, such as fearful faces, can activate subcortical structures, such as the amygdala, and engage affective and attentional networks to bias perception and cognition (Diano et al. 2017; Lapate et al. 2016; Schwabe et al. 2011). It has been shown that cardiac cycle effects on salient stimuli are associated with activity in the amygdala and periaqueductal gray (Gray et al. 2009; Garfinkel et al. 2014). Thus, it is likely that in the present study, cardiac signals modulated activity in subcortical regions such as the amygdala to enhance the representation of fearful faces, facilitating its breakthrough to awareness.

Another possibility is that the cardiac cycle did not enhance the processing of fearful faces per se but decreased the suppressive power of the masking stimuli, leading to the earlier detection of these faces. Interestingly, a recent study used a binocular rivalry paradigm where one stimulus was pulsing in synchrony with ec‐systole while the other was synchronized to ec‐diastole (Veillette et al. 2024). Results showed that stimuli synchronized to ec‐systole remained dominant for longer, suggesting facilitatory visual dominance during this cardiac period. The authors discussed the difficulty in knowing if these results are due to the suppression of the masked stimuli per se, as suggested in previous CFS studies (Salomon et al. 2016, 2018), or whether they are a result of facilitatory mask dominance due to increased lateral inhibition between neurons responsive to the two eyes. They propose that facilitation or suppression in the processing of a given stimulus could be dependent on the context. While in our study we also cannot be certain of whether the observed results are due to suppressive or enhancing effects of the cardiac cycle, the fact that modulation was only found for fearful faces does suggest a relative enhancement of salient stimuli during ec‐systole. That is, if there was a cardiac cycle influence on the suppression ability of the masking stimuli, we could expect both types of faces to show facilitated breakthrough. Instead, it is more likely that the cardiac cycle modulated the sensory processing of the face stimuli itself to enhance the representation of salient stimuli, that is, fearful faces, and facilitate the crossing of the awareness threshold.

Moreover, the direct comparison of the present study with those of Salomon et al. (2016, 2018) findings also supports stimuli or context dependent modulation of cardiac effects. The main difference between these studies is the salience of the stimuli used, that is, detect a simple dot vs. detect and discriminate the expression of a face. We argue that this pattern of relative inhibition or enhancement in stimuli processing reflects the proposed selective neuromodulation of arousal and salience networks by the cardiac cycle in a stimuli and context dependent way (Garfinkel and Critchley 2016).

Indeed, several studies have shown context‐dependent effects of the cardiac cycle (Azevedo et al. 2017; Arslanova et al. 2023; Yang et al. 2023; Schulz et al. 2011; Leganes‐Fonteneau et al. 2021). For example, the cardiac cycle modulated performance on a sequential priming implicit racial bias task when the context associated the faces with threat stereotypes but not when the same faces were associated with a positive stereotype (Azevedo et al. 2017). In another study, task‐irrelevant fearful faces presented at ec‐systole in a Go/No‐Go task were associated with more inhibition errors but fewer lapses of attention (Yang et al. 2023). These studies suggest that ec‐systole may facilitate attentional orienting processes and information encoding in conditions of motivational relevance.

Notably, this facilitation did not translate into improved accuracy or confidence in the ability to discriminate the facial expressions, suggesting no influence in the subjective evaluation of stimuli. It could have also been reasonable to expect that in conditions of heightened representation of physiological arousal (i.e., at ec‐systole) participants would be biased towards expecting a fearful face. Indeed, research has shown that expectations can modulate the CFS of emotional faces (McFadyen et al. 2022; Capitão et al. 2014). However, unlike in a previous study tapping more into attentional processes (Azevedo et al. 2017), we did not find cardiac cycle modulation of response bias, showing that cardiac signals did not influence the tendency to answer “fear” over “neutral” expression. Similarly, no modulation in the ability to discriminate between the two emotions, as indexed by d', was found. Together, these null effects suggest that, in the context of this paradigm, cardiac signals did not influence the quality of the visual percept and only modulated early sensory processes associated with stimuli access to consciousness.

Interestingly, participants were in general more inclined to identify faces as neutral, which may help to explain the relatively low accuracy in the identification of fearful faces. This may be related to the fact that instructions explicitly emphasized the need to press a key as soon as a face broke through suppression, prioritizing early sensory detection instead of the decisional processes associated with emotional discrimination. Moreover, the fact that, unlike in other CFS studies, face presentation was intermittent may also have led to brief and relatively weak visual precepts hindering the ability to consciously identify features of facial expressions of fear. In other words, the fleeting and degraded visual percept due to masking may have often lacked clear identifiable emotional features, leading participants to favor a neutral judgment over fear.

It is also interesting to note that while accuracy in the identification of fearful expressions was lower, as participants were biased towards neutral judgments, confidence for fearful faces was higher. This pattern suggests a cognitive misalignment, whereby salience and/or arousal may inflate confidence and decrease metacognition. It is difficult to pinpoint the reason for this, but it could simply reflect higher confidence when the distinctive features that characterize fearful expressions (vs. the more ambiguous features of neutral expressions) were detected. Alternatively, it could be related to the effects of arousal on confidence and metacognition (e.g., Allen et al. 2016; Geurten and Lemaire 2024; von Mohr et al. 2024), such that arousal induced by the (pre‐conscious) perception of fearful faces increases confidence irrespective of objective performance (Allen et al. 2016). Future research should further investigate this issue.

## 5 Limitations

This study has several limitations. Firstly, methodological constraints limit the generalizability to other perceptual contexts. Specifically, the need to establish cardio‐visual synchrony meant that stimuli presentation was intermittent and not continuous as in typical CFS paradigms. This, together with the emphasis given to reaction times, at the expense of emotion discriminability, may explain why accuracy in this task was lower than what is typically observed in other CFS studies. The intermittent nature of face presentation in this study also precludes more sophisticated analyses of RTs, such as Diffusion Drift Modeling, to further unveil the neurocognitive mechanisms underlying the observed effects (McFadyen et al. 2022). This is because RTs in the present task reflect face detection and do not index the timing of emotion discriminability. Secondly, we observed facilitated face detection in the (upper) visual field, but it is difficult to be certain of the reasons behind this result. It could potentially be related to differences in threat detection in different visual fields (e.g., Carretié et al. 2020) or to CFS‐related artifacts. Future research can explore this further. Thirdly, we only screened for history of cardiovascular or neurological conditions, but there is some evidence that other (mental health) conditions, notably schizophrenia, may show altered effects of cardiac afferent signaling on fear perception that can influence results (Critchley et al. 2019). Also, a few studies found that interoceptive abilities or heart rate variability modulate cardiac cycle effects (e.g., Sel et al. 2017; Suzuki et al. 2013; Gray et al. 2009). Thus, we cannot rule out the presence of confounding (e.g., psychiatric conditions or medication) or mediating (e.g., interoceptive abilities) factors explaining the observed findings. Finally, because we only used fearful expressions as examples of salient/emotional stimuli, it is not clear whether the cardiac cycle effects observed are specific to threat‐signaling stimuli (Garfinkel and Critchley 2016) or reflect broader modulation of salient/motivationally relevant stimuli. Future studies using a wider array of stimuli or facial expressions may elucidate this issue.

## 6 Conclusion

The present study expands this literature by demonstrating cardiac modulation at early visual processing stages, as indicated by facilitated breakthrough to awareness of emotional stimuli in a CFS paradigm. Interestingly, this influence does not necessarily lead to improved perceptual discrimination or metacognitive sensitivity. Together, these results extend our understanding of how this channel of body‐to‐brain communication contributes to the preconscious processing of incoming visual threat‐signaling information. Future research could make use of established neuropsychological models, such as blindsight and hemispatial neglect patients (Tamietto and de Gelder 2010; Diano et al. 2017), to further unveil the neurocognitive mechanisms underlying the cardiac modulation of sensory and attentional processes of the non‐conscious perception of emotional stimuli.

## Author Contributions

**Ozan Cem Ozturk:** conceptualization, methodology, data curation, project administration, writing – original draft, writing – review and editing, investigation. **Jessica McFadyen:** methodology, writing – review and editing, conceptualization. **Ruben T. Azevedo:** conceptualization, methodology, software, writing – original draft, writing – review and editing, supervision, funding acquisition, formal analysis, data curation.

## Conflicts of Interest

The authors declare no conflicts of interest.

## Data Availability

The data that support the findings of this study are openly available in OSF at https://osf.io/f6btp/.
